# Cancer-Related Fatigue: Causes and Current Treatment Options

**DOI:** 10.1007/s11864-020-0707-5

**Published:** 2020-02-05

**Authors:** Melissa S.Y. Thong, Cornelis J. F. van Noorden, Karen Steindorf, Volker Arndt

**Affiliations:** 1 Unit of Cancer Survivorship, Division of Clinical Epidemiology and Aging Research, German Cancer Research Center (DKFZ), P.O. Box 101949, 69009 Heidelberg, Germany; 2 Department of Medical Biology, Amsterdam University Medical Centers, AMC, Amsterdam, Netherlands; 3 Department of Genetic Toxicology and Tumor Biology, National Institute of Biology, Ljubljana, Slovenia; 4 Division of Physical Activity, Prevention and Cancer, German Cancer Research Center (DKFZ) and National Center for Tumor Diseases (NCT), Heidelberg, Germany

**Keywords:** Cancer-related fatigue, Cytokines, Physical activity, Pharmacologic, Complementary and alternative medicine

## Abstract

Cancer-related fatigue (CRF) is a problem for a significant proportion of cancer survivors during and after active cancer treatment. However, CRF is underdiagnosed and undertreated. Interventions are available for CRF although there is no gold standard. Based on current level of evidence, exercise seems to be most effective in preventing or ameliorating CRF during the active- and posttreatment phases.

## Introduction

Fatigue is a symptom commonly experienced by survivors of cancer through all stages of the disease trajectory. Survivors identify fatigue as a significant problem which is not adequately addressed by healthcare providers [1•]. Being fatigued has a greater negative impact on functioning and health-related quality of life (HRQoL) than other symptoms such as pain or depression [2, 3]. Fatigued survivors are more likely to have reduced employment participation [4, 5], greater financial stress [6], and higher healthcare utilization [6, 7]. Moreover, fatigue may reduce survival; feeling fatigued at diagnosis [6, 8] and during survivorship [9] is associated with higher mortality.

This review aims to provide a summary on the current state of research on cancer-related fatigue (CRF) of survivors with local disease treated with curative intent. We briefly summarize the prevalence, definition, evaluation, and etiology of CRF. Due to the volume of research on CRF treatments, we provide a non-exhaustive overview of treatments for CRF published within the last 5 years (guidelines, meta-analyses, reviews, randomized trials).

## Prevalence of CRF

Cancer and its treatments are often the main triggers of fatigue, either induced directly or indirectly by associated toxicities [10]. The prevalence of CRF varies, depending on whether it is assessed during active treatment or after treatment is completed. Most survivors of cancer, defined as all individuals with a cancer experience from the time of diagnosis through to the balance of his or her life by the US National Coalition for Cancer Survivorship [11], experience CRF during active treatment, which often peaks toward end of active treatment and diminishing thereafter [6, 12-15]. Nevertheless, a significant proportion of survivors who are disease-free still feel fatigued years after active treatment had ended [16, 17].

### Active or maintenance treatment phase

Among survivors undergoing active treatment, CRF rates vary between 62% and 85%, of which 9% to 45% reported moderate-to-severe CRF [18, 19]. Survivors on maintenance therapy such as adjuvant endocrine therapy or androgen deprivation therapy (ADT) are more likely to experience CRF [20, 21]. Fifty-six percent of survivors of breast cancer receiving aromatase inhibitors report moderate-to-severe CRF [22]. Survivors of prostate cancer who currently use ADT or had used ADT in the past were more likely to report CRF than survivors who never used ADT (38% current vs 23% past vs 16% never, *p* < 0.0001) [23].

### Survivorship phase

Prevalence rates of CRF also vary during the survivorship phase, depending on the time since diagnosis, short-term (< 5 years post-diagnosis) or long-term (≥ 5 years post-diagnosis).

#### Short-term CRF

Sixty percent of survivors of breast cancer reported moderate-to-severe tiredness 12 months after diagnosis [24]. Between 21% and 52% of survivors still experience severe CRF up to 3 years post-diagnosis [18, 25, 26].

#### Long-term CRF

During long-term survivorship, a significant proportion of survivors report being fatigued. One in four survivors of adolescent and young adult (AYA) cancers is still chronically fatigued 5-30 years after diagnosis [27]. Rates of chronic CRF ranging between 23% and 49% have been reported by long-term adult survivors of cervical [28], lower gastrointestinal [17], breast [29], lymphoma [30], and mixed cancers [31]. Constant levels of fatigue have also been observed from 5 to 15 years post-diagnosis in survivors of breast, colorectal, and prostate cancer [32].

### Age-related CRF

The perceived severity of CRF differs significantly by age. Intuitively, we expect fatigue to be more prominent among the elderly, with or without cancer [33]. However, the burden of fatigue seems to be greater among younger survivors of cancer when compared with non-cancer controls of comparable age [34]. The prevalence of fatigued survivors of AYA cancers is more than double that of age- and sex-matched peers [35], with 85% survivors of AYA cancers reported experiencing fatigue in the past month [36]. AYA survivors of sarcoma were more likely to report daytime CRF compared to middle-aged or elderly survivors [37]. AYA and adult, but not elderly, survivors of thyroid cancer reported clinically relevant higher levels of fatigue than non-cancer controls [38].

## Definition

The most comprehensive and commonly used definition, from the National Comprehensive Cancer Network (NCCN), describes CRF as “a distressing, persistent, subjective sense of *physical*, *emotional*, and/or *cognitive tiredness or exhaustion* related to cancer or cancer treatment that is *not proportional to recent activity* and interferes with functioning” [39••]. Of note, this definition acknowledges the multiple dimensions of CRF, and that CRF is often not relieved by rest unlike fatigue experienced by healthy individuals.

There is debate whether CRF is the expression of different dimensions of one symptom (multidimensional concept) or that it comprises separate symptoms collectively called fatigue (multiple symptom concept) [40, 41]. The heterogeneous relationship between mental and physical fatigue, each having different correlates, suggests that these symptoms are separate phenomena, giving credence to the multiple symptom concept of CRF [42, 43]. The challenge of adopting a standard definition of CRF needs to be resolved as it can have implications for the development of treatment guidelines and research into CRF [44•].

## Evaluation

Despite its prevalence and debilitating nature, CRF is underdiagnosed and undertreated [18, 45]. Up to 50% of survivors reported not discussing, getting advice, nor receiving desired help for CRF [18, 36, 46], even though clinical guidelines recommend systematic screening and management of CRF from diagnosis through to follow-up [47]. For example, the NCCN guidelines outline four general steps involving screening, evaluation, intervention, and reevaluation of CRF [39••]. Screening for CRF should be implemented for all survivors of cancer, and it can be conducted at any phase of the cancer trajectory. Survivors positively screened for CRF should then undergo a more comprehensive history and physical examination, encompassing clinical information (e.g., disease status, treatment received) and details on CRF such as the onset, pattern, duration, alleviating factors, and interference with function. Detailed evaluation of CRF is necessary not only for planning of treatment but also to rule out depression. Fatigue is a feature characteristically associated with depression [48, 49]. Interventions for CRF include pharmacologic and non-pharmacologic options. Reevaluation for CRF is recommended to assess the efficacy of the management strategy. This 4-step process is best conducted periodically, at first visit, during active treatment, after completion of treatment, and during follow-up.

### Self-reported questionnaires

As CRF is considered to be a subjective phenomenon, it is appropriate to use self-report questionnaires for assessment [39••]. Significant discrepancies in ratings of CRF severity have been observed between physician and survivor reports, with physician ratings much lower than that of the survivor [50]. For screening purposes, a valid and reliable instrument such as a numeric score with determined cutoffs is an option [39••]. Commonly used uni- or multidimensional questionnaires include the Brief Fatigue Inventory, European Organization for Research and Treatment of Cancer (EORTC) Quality of Life Questionnaire Core-30 fatigue subscale, Functional Assessment of Cancer Therapy - Fatigue, Piper Fatigue Scale [51, 52]. The EORTC has recently developed a questionnaire module that specifically assesses multiple dimensions of CRF (physical, cognitive, emotional) [53].

### Barriers to implementation of clinical management of CRF

Barriers at survivor and clinical levels contribute to the underdiagnosis and undertreatment of CRF [39••]. Survivors of cancer may perceive that CRF is inexorable, something they “have to live with” and only 19% believe that “something could be done” about CRF [36]. Often, survivors do not discuss CRF with their physician for a variety of reasons; they think CRF is unimportant; they fear either dose reduction or treatment discontinuation if they mention that they feel fatigued [47]. Although 41% of survivors have not been recommended CRF treatments [36], of those who have received either pharmacological or non-pharmacological treatments, 52% perceived that treatment was effective [18]. Likewise, clinicians may not initiate a discussion on CRF, either having perceptions similar to that of the survivors [39••] or due to lack of resources [54•].

## Mechanisms underlying CRF

Despite its prevalence and debilitating effects, the etiology underlying CRF is still not well-understood. Evidence suggests the involvement of complex multifactorial processes linked to a range of molecular/physiological, clinical, and psychological factors.

### Molecular/physiological factors

Potential disruptions originating in the central (inflammation, hypothalamic-pituitary-adrenal axis) and/or peripheral (e.g., reduced energy metabolism) nervous systems may induce CRF [44•]. Central fatigue can be characterized as a perceived reduced ability to complete physical or mental tasks without demonstrable cognitive or motor deficiencies [55, 56]. Peripheral fatigue is ascribed as the inability of muscles to perform a task in response to stimulation (“muscle fatigue”) or reduced endurance [44•, 57•].

#### Inflammation and immune system

CRF often co-occurs with symptoms such as pain, insomnia, lethargy, altered mood, and cognitive impairment, which are similar to symptoms collectively known as “sickness behavior” [58•, 59]. Sickness behavior is an adaptive response to inflammation, and there is sufficient evidence of the role of inflammation in CRF [44•, 60]. Cancer and its treatments can activate the peripheral pro-inflammatory cytokine network, producing symptoms of CRF via the cytokine signaling in the central nervous system [61•, 62]. Neuroinflammation has been postulated as a possible mechanism for persistent CRF [58•], after cessation of chemotherapy [63].

#### Hypothalamic-pituitary-adrenal (HPA) axis

Related to the activation of the inflammatory response and the immune system is the dysregulation of the HPA axis in the direct or indirect development of CRF [44•]. Cytokines released as a result of the cancer and/or its treatments into the central nervous system can stimulate the HPA axis [58•]. One function of the HPA axis is to regulate the release of cortisol during times of stress. Cortisol can inhibit cytokine production to protect the body from overactivation of the immune system and can minimize tissue damage due to inflammation [44•]. Dysregulation of cortisol levels in the blood has been associated with physical fatigue in survivors of breast cancer [64]. Altered cortisol levels have also been associated with disruption of the circadian rhythm and sleep deficiency [65•]. Flattened circadian rhythms [66] and disturbed sleep [67] during chemotherapy have been associated with CRF in survivors of breast cancer.

#### Reduced energy metabolism

A disrupted energy metabolism (adenosine triphosphate (ATP) production), as a consequence of cachexia or damage of the sarcoplasmic reticulum and/or mitochondria after chemotherapy or radiotherapy, can cause possible long-term side effects to all tissues and in particular to skeletal muscle [44•, 58•]. This effect on skeletal muscle could, in turn, increase the risk of peripheral fatigue.

### Clinical factors

#### Comorbidity

A significant proportion of survivors have problems with fatigue even before the start of treatment [68, 69], and this problem can persist [50] or even exacerbate during follow-up [14]. Pre-existing comorbid conditions such as anemia, cardiac conditions, diabetes mellitus, or psychiatric conditions could contribute to fatigue severity at the start of cancer treatment [69, 70]. Medications to treat these conditions (e.g., beta-blockers, antidepressants) could also increase fatigue [39••]. Comorbid conditions are also associated with CRF in long-term survivorship [9, 71].

#### Cancer treatment

Treatment for cancer such as surgery [72], chemotherapy [73, 74], hormone therapy [75], endocrine therapy [76], and radiotherapy [77] can result in CRF, either as a direct consequence or as a secondary effect. Of interest, although radiotherapy increased CRF in some cancers [78-80], it does not seem to be an associated risk factor in breast cancer [15, 73]. Cumulative effects of treatment and CRF are possible because survivors who received trimodal (surgery, chemotherapy, and radiotherapy, with or without hormone therapy) treatment reported higher levels of CRF than survivors receiving other treatment combinations [17, 73]. Conditions consequent to cancer treatment such as anemia [75], hypothyroidism [81], insomnia [2], pain [2], and hypopituitarism [80] could also contribute to CRF. Clinicians should be aware of and manage potentially preventable treatment-related toxicities so as to reduce the burden of CRF.

### Psychological factors

Psychological factors such as a history of depression or psychosocial distress at baseline have been associated with CRF [68, 82]. Survivors who have a tendency toward negative exaggeration and attention to symptoms (“catastrophizing”) were more likely to be more fatigued [83]. Fear of recurrence of cancer is also associated with CRF [34].

## Treatment options for CRF

Pharmacological and non-pharmacological treatments are currently used to manage CRF. However, these interventions yield, at most, moderate benefits in meta-analyses.

### Pharmacologic

Although pharmacologic treatments have been used to treat CRF, results from a meta-analysis by Mustian et al. suggest that such treatments, in comparison with non-pharmacologic (exercise and psychological) interventions, are the least effective for CRF [84••].

#### Stimulants

The rationale for using stimulants for the management of CRF is its potential rapid effect to counteract the feelings of low energy, lowered mental capabilities, or lethargy associated with fatigue [85•].

Methylphenidate, originally indicated for attentional deficit disorder, has been extensively studied for its efficacy to reduce CRF. Although clinical studies suggest that methylphenidate is superior to placebo, results from meta-analyses indicate modest effects backed by weak evidence [86, 87, 88••, 89]. Methylphenidate is generally well-tolerated. Nevertheless, it has been associated with nonserious adverse side effects such as sleep problems or decreased appetite, which could exacerbate CRF [90]. It is also contraindicated in cases of uncontrolled hypertension or cardiac problems [85•]. As such, methylphenidate should not be routinely prescribed for CRF management but selectively used for survivors whose benefits from its use outweigh the disadvantages [88••].

Modafinil is a wakefulness-inducing compound [91]. However, meta-analyses suggest that modafinil was no better than placebo in ameliorating CRF [86, 88••, 92].

Corticosteroids are not associated with reduction in CRF [88••].

#### Antidepressants

Research on the use of antidepressants in managing CRF is limited [88••]. Bupropion could be helpful for CRF when it co-occurs with depression, although it should be administered with care to survivors with anxiety problems [85•, 93•].

#### Erythropoietin

Erythropoietin can be effective in ameliorating CRF if it is associated with anemia (either at the start of or during active cancer treatment) by increasing levels of hemoglobin [88••]. However, current clinical guidelines recommend erythropoiesis-stimulating agents as treatment for anemia for only selected patients due to safety concerns regarding tumor growth and increased risk of cardiovascular events [94•, 95•].

### Non-pharmacologic

Non-pharmacologic treatments for the management of CRF during active treatment phase are more widely accepted, with a choice of options. There is ample evidence from clinical trials, systematic reviews, and meta-analyses attesting to their efficacy, to varying degrees.

#### Exercise/physical activity

Although some uncertainty still remains about the benefits of exercise in reducing CRF in general, there is compelling evidence that it does not increase CRF [96•]. A meta-analysis reported that exercise during adjuvant radiotherapy for breast cancer was more effective in reducing fatigue than a control intervention [97•]. Similarly, Hilfiker et al. showed that exercise during active cancer treatment was associated with reduced CRF [98•]. In their meta-analysis, Mustian et al. compared the effectiveness of pharmacologic, psychological, and exercise treatments for CRF. They reported that exercise interventions showed the greatest effect in reducing CRF, albeit of moderate efficacy [84••]. A Cochrane review concluded that aerobic exercise showed moderate level of evidence in reducing CRF in adult survivors of hematological cancers [99••]. In another meta-analysis, Kessels et al. reported a large pooled effect of exercise on CRF [100•].

There is increasing clinical and empirical support to provide interventions in the preoperative phase (“prehabilitation”) to optimize functional status and reduce the incidence or severity of postoperative complications [101, 102]. These interventions are often multimodal, incorporating exercise/physical activity, psychological, and nutritional aspects to enhance postoperative functioning [103]. However, these interventions are developed not specifically to reduce CRF. A study from 2011 reported on an education intervention provided prior to radiotherapy to enhance self-management of CRF [104]. Although the intervention showed no effect in reducing CRF, it did increase physical activity participation. Despite its promise in reducing postoperative morbidity and improving physical functioning, efficacy of prehabilitation programs still lacks strong evidence and may not be suitable for all survivors [105, 106].

Regarding the mode of exercise intervention (e.g., aerobic, strength/resistance, or combination of both), results on efficacy against CRF are mixed [107]. In Mustian et al.’s meta-analysis, mode of intervention was not associated with intervention effectiveness [84••]. However, in other meta-analyses, aerobic exercise (alone or in combination) was associated with large effects in reducing CRF [97•, 100•], during and after cancer treatment [98•]. A meta-analysis of randomized controlled trials of aerobic exercise and CRF suggest that exercise of longer duration can have a significant effect on CRF, especially if provided after completion of adjuvant therapy [108•].

Group-based or in-person/supervised exercise programs [84••, 100•, 107, 108•] may have an added benefit of facilitating engagement and improving adherence to the intervention [107]. In studies with high adherence, a large effect size of exercise on CRF was noted [100•].

Assessment of the long-term efficacy of exercise, post-intervention, is hampered by the generally short period of follow-up. However, evidence suggests that the positive effects of exercise could be maintained in the long term. A randomized controlled trial, involving an 18-week supervised intervention among survivors of breast and colon cancer, reported that at 4-year follow-up since start of intervention, survivors in the intervention arm reported higher levels of moderate-to-vigorous physical activity levels [109]. Although levels of physical fatigue were lower in the intervention arm, this difference was not significant.

#### Psychological/psycho-education

Psychological interventions have been shown to be modestly effective in reducing CRF, second to exercise interventions [84••].

Of the psychological interventions available, cognitive behavioral therapy (CBT) was the most effective in reducing CRF, especially if provided after end of active treatment [84••, 98•]. Furthermore, CBT has been shown to be effective in addressing insomnia, a symptom commonly coexisting with CRF, thus reducing CRF [92].

Mindfulness-based interventions are designed to enhance the emotion-focused coping of uncertainties and anxieties resulting from cancer and treatment [110]. Results from two meta-analyses and a Cochrane review indicated its effectiveness in reducing CRF in the short term, in comparison with usual care [111•, 112•, 113••]. Medium-term effects of such interventions on CRF were not significant. Most studies have focused on survivors of breast cancer [112•].

As a significant proportion of survivors of cancer may have erroneous perceptions about CRF that prevents them from seeking help [36, 39••], educational interventions could be an effective option to address these possible misconceptions. However, a Cochrane review concluded that educational interventions may have a small effect in ameliorating CRF intensity and interference on daily life and a moderate effect on reducing CRF distress [114•]. The review also recommended that efficacy of educational interventions may improve, if provided in combination with other interventions.

Mixed modal interventions (e.g., exercise and psychological or psycho-education), delivered after completion of active treatment, have been shown to be effective in reducing posttreatment CRF [84••, 98•].

#### Mind/body wellness training

A plethora of interventions aimed at improving mind/body wellness and reducing CRF is currently available, although they may not be efficacious.

Yoga is a mind/body practice of South Asian origin and is increasingly popular for the management of cancer-related symptoms. Nevertheless, clinical evidence of its effectiveness in managing CRF is equivocal. A Cochrane review indicated that there is a moderate level of evidence supporting the short-term benefits of yoga in reducing CRF when compared with either no treatment or psychosocial/educational interventions [115••]. A review of results from randomized controlled trials suggests that yoga can be effective in reducing CRF during treatment and posttreatment [116]. Clinical guidelines have upgraded the level of evidence for yoga to a “C” as a management option for posttreatment CRF [117••]. However most studies have been conducted on survivors of breast cancer and have methodological issues which need to be addressed in future studies [116].

Qigong and tai chi have also been advocated for the reduction of CRF. Both forms of intervention are rooted in Eastern traditional medical practice. Therapeutic qigong has a theoretical foundation in supporting/strengthening the psychoneuroimmunologic system [118]. The practice focuses on the restoration, rejuvenation, and healing of the body through a series of rhythmic and flowing movements including breath regulation and mindful meditation. Although tai chi has its foundation in the martial arts, when performed for therapeutic purposes, it can be considered as a form of qigong [118]. Using Bayesian network analysis, two meta-analyses found no association between qigong/tai chi and reduction in CRF [98•, 119]. Two recent meta-analyses concluded that qigong/tai chi shows promise in reducing CRF and improving HRQOL, but evidence of its efficacy needs to be strengthened [120•, 121•]. A meta-analysis of randomized controlled trials on tai chi reported that the intervention was effective in reducing CRF during the period of intervention [122]. However, long-term post-intervention effect on CRF is unclear. Future studies involving larger and heterogeneous samples, better designed with sound methodology, and longer follow-up are needed.

Acupuncture for the management of CRF has shown mixed results. Duong et al. reported in their meta-analysis that acupuncture was not effective in reducing the severity of CRF in survivors of cancer and recipients of hematopoietic stem cell transplantation [111•]. Likewise, a meta-analysis using Bayesian network analysis ranked acupuncture as the lowest in its efficacy in reducing CRF [119]. On the other hand, in their meta-analysis of 10 studies conducted mainly among survivors of breast and gynecological cancers, Zhang et al. concluded that acupuncture could be an effective option in the arsenal of CRF interventions [123]. In the clinical guidelines on integrative therapies, acupuncture for treatment of CRF had a classification of low strength of evidence and may be considered as an option for improving posttreatment CRF [117••].

Although massage has been promoted to relieve CRF, there is a paucity of evidence regarding its efficacy against CRF [111•]. Massage was not mentioned as a potential therapy for CRF in the guidelines on integrative therapies [117••]. A 2016 Cochrane review on the efficacy of massage on cancer-related symptoms including CRF concluded that there was no evidence of clinical effectiveness of massage to ameliorate CRF [124•]. In contrast, a meta-analysis in 2018 suggests that massage, given during active treatment, was effective against CRF [98•]. A recent randomized single-blind study with 66 posttreatment survivors of breast cancer reported that a 6-week Swedish massage intervention was significantly superior to light touch or waitlist control in reducing CRF [125].

Relaxation techniques which can include progressive muscle relaxation, self-hypnosis, or deep breathing have been shown to be effective in reducing CRF, [111•, 117••] especially during cancer treatment [98•]. Nevertheless, the level of evidence for relaxation techniques is low, and clinical guidelines recommend that hypnosis (self-induced or facilitated by a specialist) may be considered for reducing CRF during cancer treatment [117••]. A 12-week supervised group intervention compared the effect of resistance exercise and relaxation on CRF [126]. Results showed that resistance exercise was superior to relaxation in reducing CRF of survivors of breast cancer undergoing chemotherapy.

Reduced exposure to bright light during cancer treatment has been associated with CRF, possibly due to disruption of the circadian activity rhythm [66, 127]. Bright light therapy (BLT) is a relatively new therapy for management of CRF which requires survivors to expose themselves to bright white light in the morning for a period of time [128]. Compared with other CRF treatments such as exercise or psychological interventions, BLT is considered a safe and accessible option that requires relatively low input from survivors [129]. Results from a recent blinded randomized controlled trial were promising; the BLT group showed a 17% reduction in CRF at the end of the 4-week intervention [130]. However, research on the efficacy of BLT on CRF is limited, but another multicenter randomized trial with a longer follow-up is currently in progress [131].

#### Nutritional and dietary supplements

Healthcare providers often are not aware of the common use of nutritional and dietary supplements among survivors of cancer to manage cancer-related symptoms [132]. Clinical trials on the use of nutritional and dietary supplements to improve CRF are still limited [133•, 134]. Most studies have small sample sizes and are conducted mainly with survivors of breast cancer [135].

The most studied natural medicine to combat CRF is ginseng, comprising either the Asian (*Panax ginseng*) or American (*Panax quinquefolius*) ginseng. A systematic review on fatigue in general concluded that ginseng, regardless of type, seems to have modest efficacy in reducing fatigue although there are issues with small sample sizes and generalizability of results [136]. Another systematic review focused on survivors of breast cancer, reported mixed results from 2 studies on the efficacy of ginseng [132]. Clinical guidelines on integrative therapies rated ginseng a “C” based on the level of evidence of its efficacy in reducing CRF during active cancer treatment [117••].

Guarana is a stimulant derived from a plant native to the Amazon basin. The few studies on its efficacy to reduce CRF have small sample sizes, and results are inconsistent [132]. A double-blind crossover randomized clinical trial found no effect of guarana on fatigued survivors of breast cancer undergoing chemotherapy [137]. Based on current levels of evidence, clinical practice guidelines on integrative therapies do not recommend the use of guarana for reducing CRF during cancer treatment [117••].

Although some studies have recommended the use of L-carnitine or appetite stimulants for the management of CRF, the number of studies on these treatments are limited to allow for data synthesis in a meta-analysis [88••]. A systematic review on dietary supplements reported that L-carnitine, on its own, did not improve CRF and had associated side effects such a neuropathy and decreased functioning [135]. Based on current levels of evidence, clinical practice guidelines on integrative therapies do not recommend the use of L-carnitine for CRF reduction during cancer treatment [117••].

## Discussion

CRF is a prevalent and distressing problem for a significant proportion of survivors of cancer during active treatment and in the survivorship phase. Although there is a range of interventions available, the gold standard for the treatment of CRF remains elusive. Current levels of evidence for the treatment of CRF show, at most, moderate effects. Of the interventions available for CRF, exercise-based interventions are the most promising and are recommended as first-line treatment for CRF [84••].

It is possible that the modest treatment efficacy could be attributed to suboptimal delineation of CRF; i.e., the multidimensional nature of CRF is not considered when planning treatments [43]. As such, treatments may not be personalized to survivors’ CRF experience. It is clinically intuitive that a better classification of CRF and a more accurate identification of survivors at-risk for long-term CRF have implications for treatment response and prognosis, for example, targeting physical activity interventions to those in need, e.g., survivors with complaints of physical but not mental fatigue [138]. In contrast, survivors more burdened by mental fatigue may find relief with psychosocial interventions [12].

Although the body of research on CRF is substantial and growing, studies on interventions are still hampered by a myriad of issues such as limited heterogeneity of samples (e.g., focus mainly on survivors of breast cancer), small sample sizes, or poor methodological design with short follow-ups.
