# Correction to: Cancer-Related Fatigue: Causes and Current Treatment Options

**DOI:** 10.1007/s11864-021-00916-2

**Published:** 2022-03-01

**Authors:** Melissa S.Y. Thong, Cornelis J. F. van Noorden, Karen Steindorf, Volker Arndt

**Affiliations:** 1 Unit of Cancer Survivorship, Division of Clinical Epidemiology and Aging Research, German Cancer Research Center (DKFZ), P.O. Box 101949, 69009 Heidelberg, Germany; 2 Department of Medical Biology, Amsterdam University Medical Centers, AMC, Amsterdam, Netherlands; 3 Department of Genetic Toxicology and Tumor Biology, National Institute of Biology, Ljubljana, Slovenia; 4 Division of Physical Activity, Prevention and Cancer, German Cancer Research Center (DKFZ) and National Center for Tumor Diseases (NCT), Heidelberg, Germany

**Correction to: Curr. Treat. Options in Oncol. (2020) 21: 17**

10.1007/s11864-020-0707-5

The article "Cancer-Related Fatigue: Causes and Current Treatment Options", written by Melissa S.Y. Thong, Cornelis J. F. van Noorden, Karen Steindorf, and Volker Arndt, was originally published Online First without Open Access. After publication in volume 21, issue 2, article 17 the author decided to opt for Open Choice and to make the article an Open Access publication. Therefore, the copyright of the article has been changed to ©The Author(s) 2020 and the article is forthwith distributed under the terms of the Creative Commons Attribution 4.0 International License, which permits use, sharing, adaptation, distribution and reproduction in any medium or format, as long as you give appropriate credit to the original author(s) and the source, provide a link to the Creative Commons licence, and indicate if changes were made. The images or other third party material in this article are included in the article’s Creative Commons licence, unless indicated otherwise in a credit line to the material. If material is not included in the article’s Creative Commons licence and your intended use is not permitted by statutory regulation or exceeds the permitted use, you will need to obtain permission directly from the copyright holder. To view a copy of this licence, visit http://creativecommons.org/licenses/by/4.0/. Open Access funding enabled and organized by Projekt DEAL.

The original article has been corrected.

